# MildInt: Deep Learning-Based Multimodal Longitudinal Data Integration Framework

**DOI:** 10.3389/fgene.2019.00617

**Published:** 2019-06-28

**Authors:** Garam Lee, Byungkon Kang, Kwangsik Nho, Kyung-Ah Sohn, Dokyoon Kim

**Affiliations:** ^1^Department of Software and Computer Engineering, Ajou University, Suwon, South Korea; ^2^Biomedical & Translational Informatics Institute, Geisinger, Danville, PA, United States; ^3^Center for Computational Biology and Bioinformatics, Indiana University School of Medicine, Indianapolis, IN, United States; ^4^Center for Neuroimaging, Department of Radiology and Imaging Sciences, Indiana University School of Medicine, Indianapolis, IN, United States; ^5^Department of Biostatistics, Epidemiology and Informatics, Perelman School of Medicine, University of Pennsylvania, Philadelphia, PA, United States; ^6^Institute for Biomedical Informatics, University of Pennsylvania, Philadelphia, PA, United States

**Keywords:** multimodal deep learning, data integration, gated recurrent unit, Alzheimer’s disease, python package

## Abstract

As large amounts of heterogeneous biomedical data become available, numerous methods for integrating such datasets have been developed to extract complementary knowledge from multiple domains of sources. Recently, a deep learning approach has shown promising results in a variety of research areas. However, applying the deep learning approach requires expertise for constructing a deep architecture that can take multimodal longitudinal data. Thus, in this paper, a deep learning-based python package for data integration is developed. The python package deep learning-based **m**ult**i**modal **l**ongitudinal **d**ata **int**egration framework (MildInt) provides the preconstructed deep learning architecture for a classification task. MildInt contains two learning phases: learning feature representation from each modality of data and training a classifier for the final decision. Adopting deep architecture in the first phase leads to learning more task-relevant feature representation than a linear model. In the second phase, linear regression classifier is used for detecting and investigating biomarkers from multimodal data. Thus, by combining the linear model and the deep learning model, higher accuracy and better interpretability can be achieved. We validated the performance of our package using simulation data and real data. For the real data, as a pilot study, we used clinical and multimodal neuroimaging datasets in Alzheimer’s disease to predict the disease progression. MildInt is capable of integrating multiple forms of numerical data including time series and non-time series data for extracting complementary features from the multimodal dataset.

## Introduction

As the amount of biomedical datasets grows exponentially, the development of relevant data integration methods that can extract biological insight by incorporating heterogeneous data is required (Larranaga et al., 2006). Recently, deep learning approaches have shown promising results in numerous applications such as natural language processing, computer vision, and speech recognition. In addition, in the field of translational research, deep learning-based predictive models have shown comparable results (Chaudhary et al., 2017; Choi et al., 2017; Lu et al., 2018; Lee et al., 2019). In previous studies, they integrated multiple domains of data using deep learning models to discover integrative features that cannot be explained by a single domain of data. For example, multimodal neuroimaging dataset is combined in (Lu et al., 2018) using deep learning-based framework for discriminating cognitively normal with Alzheimer’s disease (AD), which resulted in considerable performance improvement. For the multi-omics data integration, RNA-seq, miRNA-seq, and methylation data from The Cancer Genome Atlas (TCGA) are incorporated using auto-encoder for predicting hepatocellular carcinoma survival (Chaudhary et al., 2018). Furthermore, in (Lee et al., 2019), multimodal gated recurrent unit (GRU) is used to integrate cognitive performance, cerebrospinal fluid (CSF), demographic data, and neuroimaging data to predict AD progression. Data integration is believed to help improve the classification performance by extracting complementary information from each domain of source.

However, integrating heterogeneous data is a challenging task. First of all, multimodal data might hinder learning complementary feature representation due to the presence of mutually exclusive data, that is, a useful feature representation of the data might not be learned well since the task-irrelevant portion of the data could interfere with the task-relevant portion. In addition, dealing with datasets that consist of multiple time points is another issue for data integration. Time series data include multiple time points of data, whose length is varied over samples, while non-time series consists of a single time point of data. Thus, additional transformation steps for time series dataset should be preceded to convert the variable-length sequence data into fixed-size representations without losing information. Finally, most commonly, the more various datasets are used, the less sample size is available. Traditional data integration methods use only samples overlapped by all modalities. Since only a few samples contain all modalities of data, it is inevitable to use a small portion of the samples, even though abundant samples are available.

In this paper, we provide a deep learning-based python package for heterogeneous data integration. The most significant advantage of our package is the flexibility in which irregular time series data are processed. As the main component of our package, we combine multiple GRUs with simple concatenation-based vector integration, which makes it possible to incorporate any number of modalities. Furthermore, nonoverlapping samples, as well as overlapping samples, can be used for training a classifier. To demonstrate the validity of our package, we conduct experiments on simulation data and real data. For simulation data, we generate multimodal time series data using the autoregressive model and solve a binary classification task. For the real data, as a pilot test, patients with mild cognitive impairment (MCI) is used to predict AD progression.

### Methods

**Figure 1:**
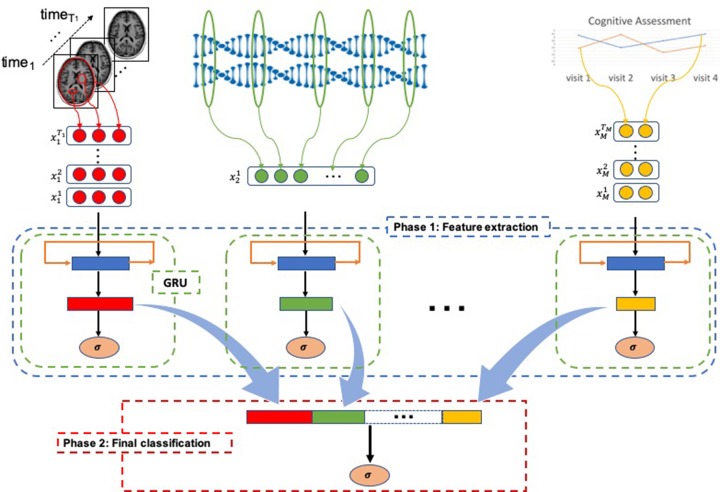
Longitudinal total intracranial volume, hippocampal volume, and entorhinal cortex thickness from brain imaging data, genomic data, cognitive assessment, and any forms of numerical data that can be taken using our framework. In phase 1 (blue-dashed rectangle), each modality of data is separately processed for learning feature representation. Both time series and non-time series data can be accepted to produce fixed-size feature vectors using a gated recurrent unit (GRU) component (green-dashed rectangle). Then, the learned representations (rectangles colored by red, green, and yellow) are simply concatenated to form an input for logistic regression (LR) classifier in phase 2 (red-dashed rectangle).

As shown in **Figure 1**, MildInt comprise two learning phases: 1) feature extraction from each modality of data and 2) learning the integrative feature representation to make the final prediction. In phase 1, time series data from a single domain is transformed into a fixed-size vector. Then, vectors from each modality of data are integrated and fed to logistic regression (LR) classifier for the final decision making in phase 2. We use GRU as our main component for learning feature representation from the time series data. Additionally, we apply the concatenation-based data integration method to integrate multiple sources of data into a single vector.

## Phase 1: Feature Extraction From Each Single Modal Time Series Data

### Recurrent Neural Network

Recurrent neural network (RNN) is a class of deep learning architecture composed of multiple recurring processing layers to learn a representation of sequential data (LeCun et al., 2015). An RNN processes an input sequence one element at a time and updates its memory state that implicitly contains information about the history of all the past elements of the sequence. The memory state is represented as a Euclidean vector (i.e., a sequence of real numbers) and is updated recursively from the input at the given step and the value of the previous memory state. Given a sequence *X* = {*x*_1_,*x*_2_,…,*x_t_*…,*x_T_*} memory state and output at each time step are computed as follows:

(1) $$s_{t}=\tanh\;(U(s)x_{t}+W^{\left(s\right)}s_{t-1}$$

(2) $$o_{t}=\mathrm{softmax}(V^{\left(o\right)}s_{t})$$

where *U*, *W*, and *V* are parameters to be learned for computing input, memory state, and output, respectively. Output is resulted from softmax function whose role is to convert the vector of hidden state into a probability vector *via* the following operation:

(3) $$\sigma (u_{i})=\frac{e^{u_{i}}}{\sum_{k}e^{u_{k}}}$$

where *u_i_* is the *i*-th element of the vector *u* and *k* is the number of labels. Finally, loss function is defined with cross-entropy to quantify the distance between true label and estimated one. In our package, only the last output *o_T_* is picked and used for the estimate because the output is regarded to carry the past features relevant to estimation.

In natural language processing, speech recognition, and anomaly detection in time series, RNN is popularly used for analyzing the sequence of words and time series data (Deng et al., 2013). One of the main advantages of using RNN is that variable length of time series data can be processed. This advantage is a critical part of our framework that is capable of accepting any variable length of time series data. However, extracting features in a long sequence of data is hard for RNN, which is known as a long-term dependency problem (Bengio et al., 1994). To handle this problem, long short-term memory (LSTM) and GRU have been developed and practically used. 

### Learning Feature Representation Using Gated Recurrent Unit

GRU and LSTM are the extension of RNN in which additional parameters regulate the memory state, making it possible to “forget” irrelevant, outdated past information. Although both LSTM and GRU can handle long-term dependency problem, we selected GRU as the main component of MildInt. Since GRU has fewer parameters than LSTM, it is expected that GRU is easier for training in the field of translational informatics where only a few samples are available. 

Regulating long-term information is handled by reset and update gates. Parameters for both gates are learned for determining how *x_t_* is processed [equation (4)–(7)]. Update gate decides how amount of the previous memory value s*_t_*_−1_ is passed on. Suppose z*_t_* is computed as 1 by equation (4), then only the previous memory is passed on, while newly computed hidden value *h_t_* will be forgotten [equation (7)]. On the other hand, reset gate manipulates the computation between previous memory *s*_*t*−1_ and the current input *x_t_*. In equation (6), reset gate determines the amount of previous memory value *s*_*t*−1_. Note that GRU is a general case of RNN because setting *r_t_* to 1 and *z_t_* to 0 for *t* = 1,2, … , *T* leads GRU to functioning exactly the same as RNN.

(4) $$z_{t}=\sigma (U^{\left(z\right)}x_{t}+s_{t-1}W^{\left(z\right)})$$

(5) $$r_{t}=\sigma (U^{\left(r\right)}x_{t}+s_{t-1}W^{\left(r\right)})$$

(6) $$h_{t}=\tanh\;(U^{\left(k\right)}x_{t}+(s_{t-1}\circ r_{t})W^{\left(h\right)})$$

(7) $$s_{t}=(1-z_{t})\circ h_{t}+z_{t}\circ s_{t-1}$$

In **Figure 1**, $x_{m}^{t}$ represents *m*-th modality of data at *t* time point. *T_m_* is the maximum time length of *m*-th modality. A single GRU takes each modality of time series data separately for learning fixed-length representation in the first phase. Note that every modality of data is assumed to be a time series data in our package. For the single time point modalities, they are also considered as length-1 time series data for ease of integration. Without multiple time points of input data, GRU is only a fully connected network with a prior hidden state. Thus, the GRU component is able to take not only time series data but also non-time series data as well. The feature representations learned in the first phase are optimized only by a single modality of data. Thus, phase 1 can be used for a feature learning phase from a single domain of source. 

## Phase 2: Final Classification

In the second phase, integration of multiple domains of data takes place. The feature representations are learned separately in the first phase. Thus, a vector produced from a GRU component contains only the information of a single modality. For learning integrative feature representation in the second phase, vectors are simply concatenated (**Figure 1**). Based on the concatenated vector, any classification algorithm can be used in phase 2. In our package, we provide LR because it yields good interpretability by analyzing beta coefficients of the trained classifier. Also, in the experiments with real data and simulation data, an LR model was used for the final decision.

LR is a classification algorithm in which the outcome is the probability of binary classes. Sigmoid function transforms the linear combination of the input features into probability values that can be mapped to the binary class. We apply *l*_1_-regularized LR for the classification. A python library Sklearn (Bengio et al., 1994) is used for LR in our package. 

## Results

To validate the performance of our package, experiments on simulation data and real data are conducted. In the experiment with simulation data, multimodal time series data are generated and tested for binary classification. The classification performance of our package is compared with other well-known methods such as logistic regression (LR), random forest (RF), and support vector machine (SVM). In the experiment with real data, four modalities of datasets, such as cognitive performance, cerebrospinal fluid (CSF), demographic data, and MRI data of patients in Alzheimer’s disease, are used for MCI conversion prediction that is also set to binary classification.

## Classification Task on the Simulation Data

In this section, we demonstrate the performance improvement using multimodal data and time series data. In the first experiment, only a single time point of data is used to evaluate the performance improvement of MildInt over other prominent classification algorithms such as SVM, LR, and RF. In the following experiment, the performance of using time series data is observed to evaluate the effectiveness of applying additional time points of data.

To generate time series data for binary classification, we apply the autoregressive model. First two underlying networks *A*_0_ and *A*_1_ are generated for the parameters in the autoregressive model. It is assumed that individual record is generated based on the underlying network in which 0-labeled data are generated from network *A*_0_ while 1-labeled data from *A*_1_. The underlying network *A*_0_ is built in which edges are randomly selected as either 0 or 1, and a network *A*_1_ against *A*_0_ is built with a distance *d* ranging from 0 to 1 in equation (8) where $A_{0_{ij}}$ is an element of the *i*-th row and the *j*-th column in the network *A*_0_ whose size is *n *× *n*.

(8) $$\begin{array}{cc} A_{1_{ij}}=\left|A_{0_{ij}}-d\right|, & for\;1\;\leq i,j\leq n \end{array}$$

The distance *d* is a value for how likely two matrices *A*_0_ and *A*_1_ are distinguishable. For example, if *d* = 1, then *A*_0_ and *A*_1_ are opposite matrices where edges in *A*_0_ are not in *A*_1_ while edges in *A*_1_ are not in *A*_0_. On the other hand, if *d* = 0, *A*_0_ and *A*_1_ are exactly the same. Thus, dataset generated with higher *d* is easier to be separated. Second, we pick up sets of nodes from the underlying network to make subnetworks. Each subnetwork is considered as each modality of data because each modality of data is assumed to have a part of information for understanding entire networks. Finally, time series data are generated using the nonlinear autoregressive model in equation (9) where M is a subnetwork and ε is an error term with 0 mean and 0.1 variance.

(9) $$\begin{array}{cc} x^{t}=\sigma (Mx^{t-1}+\varepsilon ), & \varepsilon \sim N(0,0.1) \end{array}$$

$$x^{0}\sim U(-1,1)$$

We generated 1,000 samples whose length of time points is 10. Among 1,000 samples, only 500 samples contain all modalities of data, while the rest of them have only a part of all modalities. For evaluation, we ran fivefold cross-validation 10 times in which every fold has the same ratio of positive and negative samples.

**Figure 2:**
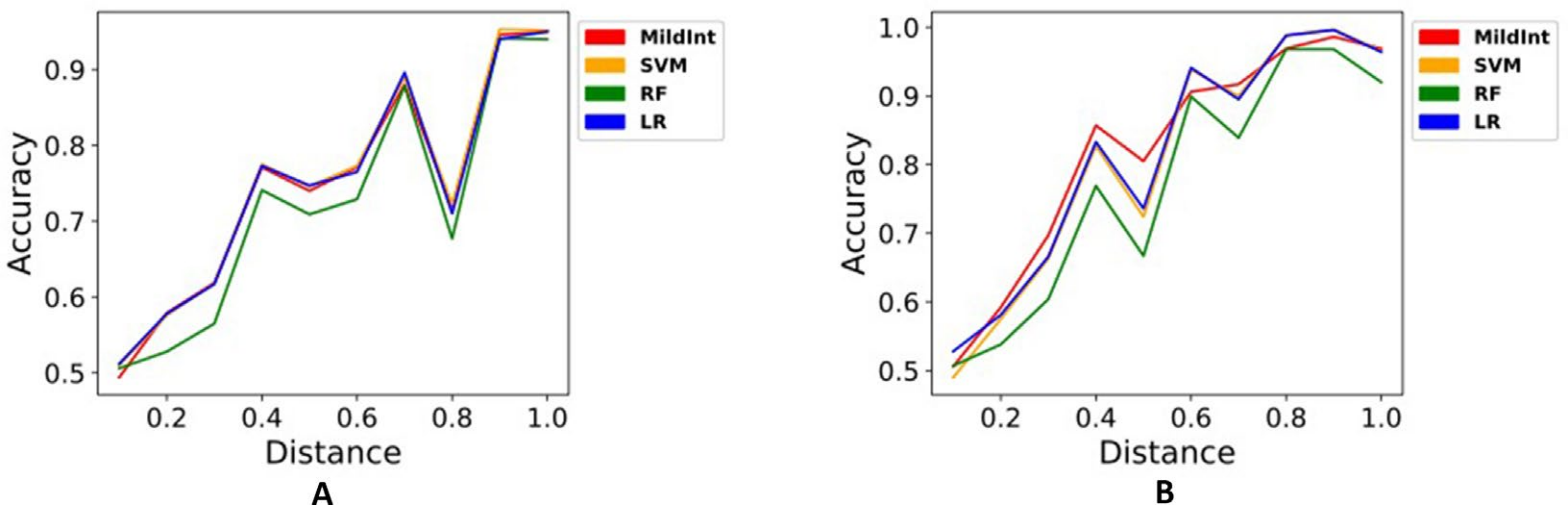
Classification performances of test set with *MildInt*, *SVM*, *random forest*, and *logistic regression* using single modality of data **(A)** and multi-modality of data **(B)**.

In **Figure 2**, we only used a single time point of data to compare the classification performance depending on modality. **Figure 2A** shows inconsistent accuracies of SVM, RF, LR, and MildInt over distances since single modality of data does not contain enough information for understanding whole underlying networks. Thus, the performance becomes more affected by the error term. Contrary to the performance with single modality, performance using multi-modality of data is less affected by error term. As shown in **Figure 2B**, accuracy is improved consistently over distances from 0.5 to 1.0. In particular, the performance of MildInt shows 1.0 accuracy over distances from 0.8 to 1.0 since MildInt can take non-overlapping as well as overlapping samples on input, while SVM, RF, and LR can only use overlapping samples.

**Figure 3:**
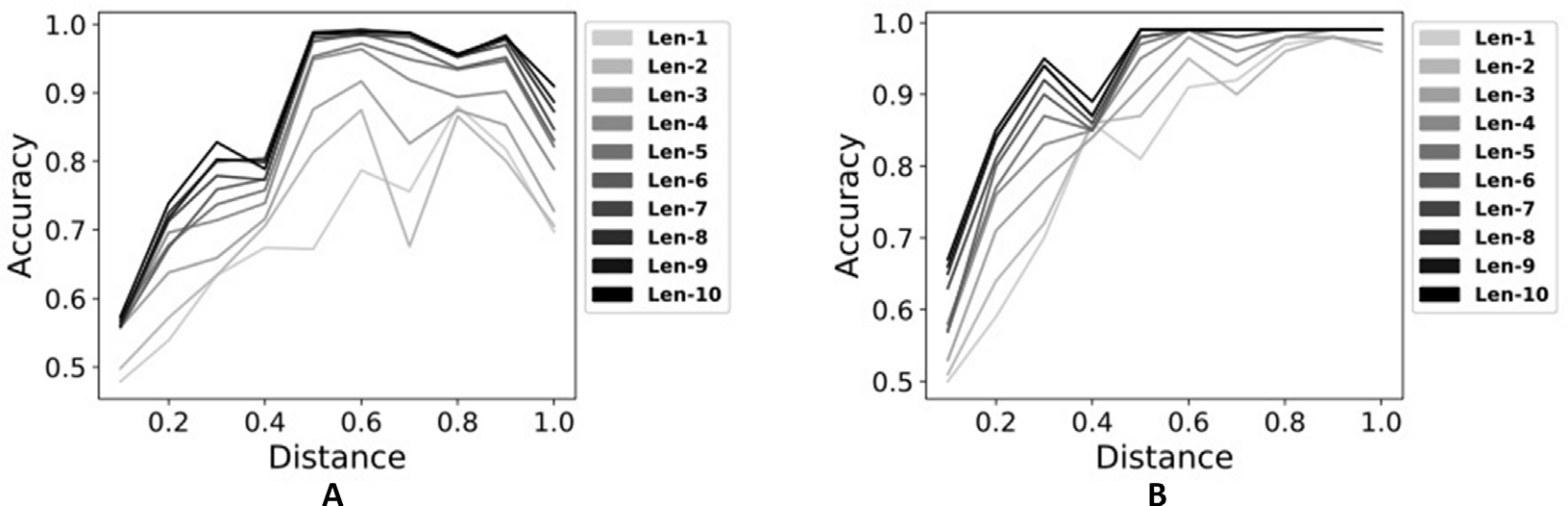
Classification performances using time series data with single modality **(A)** and multimodality **(B)**.

From **Figure 3**, we can see the effectiveness of using time series data. As increasing the number of time points, the performance using single modality is consistently increased (**Figure 3A**). Using multi-modality of time series data whose length is more than 6, two sets of data are perfectly classified from the distance 0.5 to 1.0 as seen in **Figure 3B**. Intuitively, data from multiple time points have more information than data at a single time point. Thus, MildInt can exploit temporal changes in time series data for the correct classification.

## Classification Task on the Real Dataset

For the experiment with real data, we used 865 subjects in MCI obtained from Alzheimer’s disease neuroimaging initiative cohort (ADNI) for predicting AD progression. The overall objective of ADNI is to test whether neuroimaging, biological markers, clinical, neuropsychological assessment could be combined to measure the AD progression. We downloaded four modalities of data including cognitive performance, CSF, magnetic resonance imaging (MRI), and demographic information; each of which has 802, 601, 865, and 865 samples, respectively, from the ADNI data repository (http://adni.loni.usc.edu). Informed consent was obtained for all subjects, and the study was approved by the relevant institutional review board at each data acquisition site (for up-to-date information, see http://adni.loni.usc.edu/wp-content/themes/freshnews-dev-v2/documents/policy/ADNI_Acknowledgement_List%205-29-18.pdf). All methods were performed in accordance with the relevant guidelines and regulations. Among the four modalities of samples, 601 overlapping samples are available with 200 MCI converter and 401 MCI non-converter samples. Cognitive performance and CSF are time series data with lengths of 4.05 and 1.69 on average. MRI and demographic information are considered as length-1 time series data in our package. Note that all modalities are given in numerical vector forms. For example, we extracted gender, age, level of education, and cognitive assessment from patients’ record. Especially for MRI data, a preprocessing was performed to extract features, such as total intracranial volume, hippocampal volume, and entorhinal cortex thickness, which are relevant to predicting MCI conversion. Recent methods (Lama et al., 2017; Sandeep et al., 2017) that extract features also can be used before running our package. The summary statistics of samples and hyperparameters are shown in **Table 1**.

**Table 1:** Summary statistics for data and hyperparameters in the experiment with real data.

	#Features	Hidden dimension	Time length (avg)	Time length (sd)
Cognitive performance	2	3	4.05	1.71
Demographic information	4	5	1	0
CSF	5	6	1.69	0.95
MRI	3	4	1	0

CSF, cerebrospinal fluid; MRI, magnetic resonance imaging.

**Figure 4:**
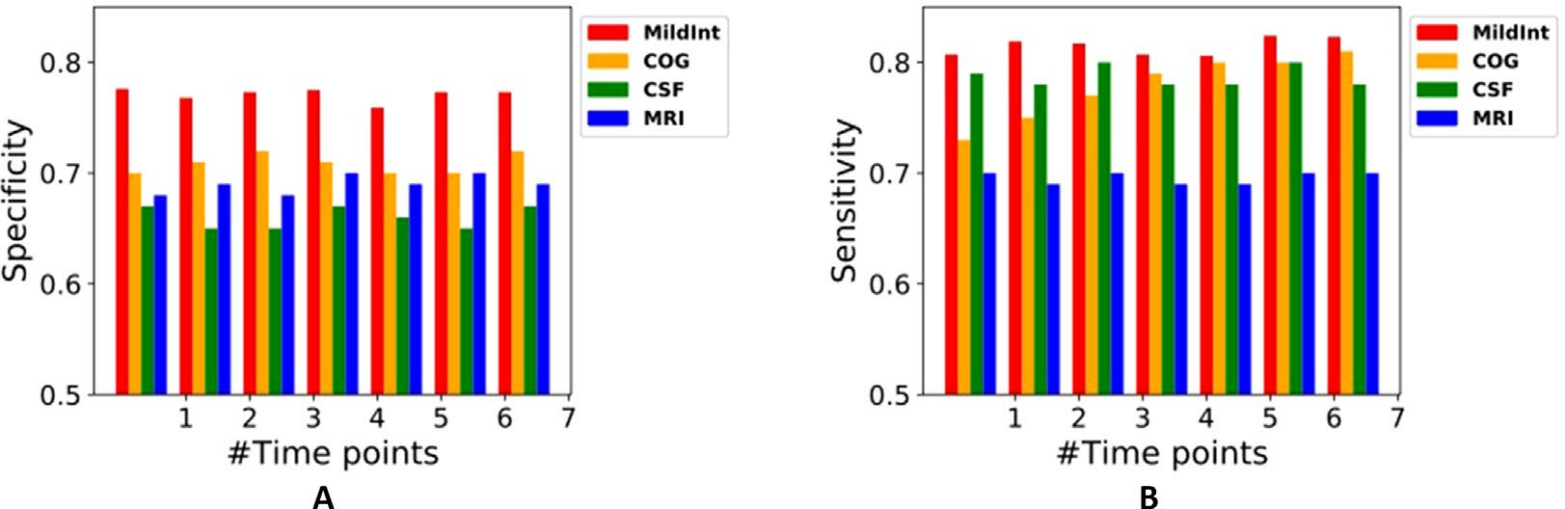
Predictive performances using multi-modality and single modality of data.

**Figure 4** shows the accuracies of our package using time series data. We removed the accuracy from the model with demographic data because the prediction performance was too low. The performance improvement using time series data is marginal due to the sparsity of time points. More than half of the samples contain missing values, and even the length of time points is short. Furthermore, we have longitudinal samples for only two modalities of data (cognitive performance and CSF). Thus, it is hardly expected that the performance is enhanced using longitudinal data. However, classification accuracy was improved using multiple domains of data. As seen in **Figure 4**, integrating four sources of data shows the best predictive performance compared with the performance with single modalities. Finally, we compared the performance of MildInt with previously developed methods for MCI conversion prediction. As observed in **Table 2**, MildInt showed comparable prediction results.

**Table 2:** A list of previous models that train classifiers mainly with mild cognitive impairment (MCI) samples.

Method	Subjects (MCI-C/MCI-NC)	Data source	ACC	SEN	SPE
SVM (Zhang and Shen, 2012a)	43/48	MRI, PET, CSF	0.73	0.68	0.73
SVM (Cheng et al., 2012)	43/56	MRI, FDG-PET, CSF	0.79	0.84	0.72
SVM (Zhang and Shen, 2012b)	35/50	MRI, PET, cognitive score	0.78	0.79	0.78
Gaussian process (Young et al., 2013)	47/96	MRI, PET, CSF, APOE genotype	0.68	**0.90**	0.52
Hierarchical ensemble (Huang et al., 2017)	70/61	MRI	0.79	0.86	0.78
Deep neural network (Lu et al., 2018)	235/409	MRI, PET	**0.82**	0.79	**0.83**
MildInt	163/376	Cognitive score, neuroimaging data, CSF biomarker, demographic data	0.79	0.83	0.77

MCI-C, MCI-Converter; MCI-NC, MCI-NonConverter; ACC, Accuracy; SEN, Sensitivity; SPE, Specificity; APOE, Apolipoprotein E; FDG; Fluorodeoxyglucose.

## Conclusion

MildInt provides multimodal GRU for heterogeneous data integration. The main advantage of our framework is that variable-length time series data and multimodal data can be processed. In addition, every available sample from all modalities including non-overlapping samples can be used for training classifier. The performance of MildInt is evaluated with simulation data and real data. In the experiment with simulation data, it showed the best performance when multimodal data and time series data were integrated. Additionally, in the experiment with real data, integrating cognitive performance, demographic information, CSF, and MRI imaging data show the best performance for MCI conversion prediction. Also, any numerical form of data such as gene expression, methylation, and single nucleotide polymorphism data can be combined in our package. MildInt is suitable to use in cases where time series data such as multiple time points of methylation data and non-time series data such as single nucleotide polymorphism should be incorporated for learning integrative feature representation. Furthermore, compared with previously developed methods, MildInt showed comparable prediction ability that can efficiently incorporate multiple domains of resources.

## Requirements

This package works on python 2.7.x in platforms such as Mac OS X, Windows, and Linux. MildInt requires python packages such as Pandas, Numpy, Tensorflow, and Sklearn to be installed independently. To make MildInt fully functioning, Tensorflow with graphics processing units (GPU) from NVIDIA should be equipped. The GPU-enabled version of Tensorflow has requirements such as 64-bit Linux, NVIDIA CUDA 7.5 (CUDA 8.0 required for Pascal GPUs), and NVIDIA, cuDNN v4.0 (minimum) or v5.1 (recommended).

## Author Contributions

This study was conceived by GL, K-AS, and DK. Experiments were designed and performed by all authors. The manuscript was initially written by GL. All the authors revised the manuscript and approved the final version prior to submission.

## Funding

The support for this research was provided by NLM R01 LM012535, NIA R03 AG054936, and the Pennsylvania Department of Health (#SAP 4100070267). The department specifically disclaims responsibility for any analyses, interpretations, or conclusions. This work was also supported by the National Research Foundation of Korea grant funded by the Korea government (MSIT) (no. NRF-2019R1A2C1006608).

## Conflict of Interest Statement

The authors declare that the research was conducted in the absence of any commercial or financial relationships that could be construed as a potential conflict of interest. 
